# The question of meaning—a momentous issue for qualitative research

**DOI:** 10.1080/17482631.2019.1598723

**Published:** 2019-04-08

**Authors:** Helena Dahlberg, Karin Dahlberg

**Affiliations:** a Institute of health and caring sciences, University of Gothenburg, Gothenburg, Sweden; b Professor Emeritus, Caring science

**Keywords:** Qualitative research, meaning, content analysis, phenomenology, method, description, interpretation

## Abstract

In this article, we identify some worrying problems in the contemporary practice of qualitative research, such as the confusion regarding content and meaning in content analysis, the frequent use of standardized methods that avoids philosophy, as well as the description/interpretation dichotomy in empirical research. Since they all arise from a failure to understand the concept of meaning, we return to the question of meaning as the axis that qualitative research pivots around. We examine the meaning of meaning, and how meaning differs from content, and we then ask what consequences this has for research. Even though our analysis is rooted in phenomenological philosophy, we argue that that the ideas that we present are valid for any qualitative research approach. The question of understanding and relating to meaning, we argue, is a momentous issue for qualitative research, where we either continue safeguarding the very essence of qualitative research as dealing with human phenomena, or give it up in favor of more pragmatic and clear-cut methods that seemingly does away with the question of meaning.

## Introduction

For some decades, qualitative research has contributed with significant research findings in several scientific disciplines. It has been successful in disclosing some of the worst positivistic misunderstandings regarding science and human being. By revealing the inadequacies of the dominant quantitative research paradigm when it comes to understanding phenomena such as health and illness, it has earned legitimacy within the enterprise of producing scientific knowledge—even though it remains in the periphery when it comes to e.g., policy decisions and ranking evident research results.

As we see it, however, qualitative research has not developed satisfactorily. In particular, we see two problems that need to be sorted out and taken care of. The first one is the confusion regarding content and meaning in content analysis. Content analysis is frequently employed within e.g., health care science, and the practice of this method conveys that, even though the title says “content”, the studies deal with “meaning” in terms of e.g., “meaning units” and “interpretation” (e.g., Graneheim & Lundman, 2004; Graneheim, Lindgren, & Lundman, 2017; Elo & Kyngäs 2008). However, the meaning of and relation between “content” and “meaning” is benighted and there is no epistemological support for how one should understand these concepts.

Content analysis is one example of a method approach within qualitative research that avoids philosophy and theory of science, and where more or less standardized method procedures are recommended (another example is Smith, 2011; Smith, Flowers, & Larkin, 2009). This kind of approaches, where philosophy is avoided in favour of more pragmatic methods, seems to become more and more common in qualitative research, and the reasons for this can be many. Philosophy is seen as too complicated, too complex, or too opaque to use as a guide for conducting research (e.g., Paley, 2017), or as simply providing a “window dressing” for a study (Thorne, 2011, 2013). These arguments are thought even more valid when it comes to guiding students in their first contact with research. The alleged obscure philosophy is thus replaced with pragmatic methods that provide simple technique and a variety of tools. Meaning analysis is replaced with content analysis.

The second problem is the description—interpretation dichotomy in qualitative empirical research. On the one hand there is a descriptive approach, often referred to as “phenomenological” (e.g., Giorgi 2009) and on the other hand there is an interpretive approach, often referred to as “hermeneutical” (e.g., van Manen, 1990). Researchers who apply these methods, we argue, tend to make things worse by using “interpretation” as a key concept in a “hermeneutic” method approach, separating it from a description of meaning (e.g., Hopkins, Regehr, & Pratt, 2017; Horrigan-Kelly, Millar, & Dowling, 2016; Mackey, 2005), thereby reinforcing the differences between “description” and “interpretation.” The continental tradition is, indeed, heterogeneous and many-faceted, and the philosophy must be thoroughly examined and discussed in order to be practised in empirical research, but this side-taking, even animosity, that we see in terms of the description—interpretation dichotomy is mostly negative. In a recent publication we have shown that the controversy is built upon several misunderstandings and that it has no ground in the referred to philosophy. As a consequence, we propose “a third way” for qualitative research with its roots in phenomenology and hermeneutics (Dahlberg & Dahlberg, 2019).

As we see it, the confusion regarding content and meaning in content analysis, the frequent use of standardized methods, as well as the description-interpretation controversy, have one thing in common. They all arise from a failure to understand the concept of *meaning*. The question of understanding and relating to meaning, we argue, is a momentous issue for qualitative research. We have now come to a decisive moment: shall we continue to safeguard the very essence of qualitative research as dealing with human phenomena, or shall we give it up in favour of more pragmatic and clear-cut methods? If we fail to address this issue as qualitative researchers, and instead turn to methods that seemingly does away with the question of meaning, or that transforms the task of understanding meaning into a fixed procedure or even a computer operation, we will indeed remove our own *raison d’être*.

In this article, we return to the question of meaning as the axis that qualitative research pivots around. We begin by examining the meaning of meaning, and how meaning differs from content. We then ask what consequences this has for research. Our analysis is rooted in phenomenological philosophy, including hermeneutics, and in our own experiences of undertaking phenomenological research, as well as advising students at all academic levels. A previously published approach to qualitative research, *Reflective Lifeworld Research* (Dahlberg, Dahlberg, & Nyström, 2008) is also part of our framework. Our intention, however, is that the idea that we present is valid for any qualitative research approach.

## Content and meaning

How then, can we understand the difference between content and meaning? Let’s say that we are doing an interview, and that the person in front of us states “I’m in pain.” The content of this statement is pretty straightforward; the person is in pain. But was does this statement *mean*? To answer this, we need more information about the context of the statement. It might be, for example, that the person is speaking about physical pain in his neck after a traffic injury, or about the pain that he feels after his wife left him. It might also be that he speaks about a physical pain in order to express a psychological pain that is too hard or too complex to put into simple words. Or perhaps the expression of pain is an attempt to elicit feelings of sympathy from the interviewer. To experience pain can also mean very different things for different persons, in different circumstances. For an elderly woman, the fact of having pain might mean that she cannot go on her daily walk, which used to bring her the pleasure of fresh air, nature and exercise. For a writer, a recurring pain might make it impossible to concentrate on her professional work. For a man that always took pride in providing for his family, the fact of being in pain might bring with it shameful feelings of being weak, of not being able to do what is expected of him. For a woman giving birth, the pain might instead be a positive experience that with each labour brings her closer to her child.

The meaning of an expression is thus not self-evident, but relates to the person and the context in which it is being expressed. In an interview situation where we are intent upon finding out the meaning of pain, we thus need to ask follow-up questions that reveal what this person means when talking about pain, what kind of pain there is and what these experiences mean to the person. In the process of analyzing our empirical material, we have to ask similar questions to the text: What is the meaning of this statement? What is the meaning of this statement in the context of the whole interview? In the context of all interviews?

## The meaning of meaning

Understanding meaning is easy and uncomplicated, and at the same time difficult and challenging. It is easy and uncomplicated, even unavoidable, in our everyday way of relating to each other and our world, but challenging when we have to use this habitual ability in research practices. With the assistance of Merleau-Ponty’s philosophy, we will now explicate the very mundaneness of meaning, before we delve into the challenges that this mundanity presents us with when doing research.

“We are condemned to meaning [*nous sommes condamnés au sens*].” With these words, Merleau-Ponty (1995, p. xxii, 1945, p. xiv) displays that as human beings, we cannot choose to engage with meaning or not, but rather, meaning is unavoidable. It is part of our existence, of how we relate to our world and others. The French word that Merleau-Ponty most often employs to speak of meaning is *sens—*equivalent to the English word *sense*. The rich meaning of the word *sens/sense* indicates in what way meaning is a fundamental part of our existence. We can for example speak of the “sense of a word” or of how to “make sense” of something, pointing at the meaning of a word or event that we usually grasp intuitively. When I exclaim “Now it makes sense to me!” it means that I have understood the significance or rationale of something that before was unintelligible or incoherent. That which before seemed like separate and muddled pieces joins together and forms a coherent whole. But *sens/sense* is also closely connected to the senses and their ability to grasp the world—we can “sense” the smell of roasted coffee beans, and we can “sense” that someone is angry. Perception can thus be understood precisely as “sensing”, that is, as understanding meaning. With reference to Husserl’s understanding of intentionality, Merleau-Ponty (1995, 1945) explains that perception is always the perception *of something*, i.e., the grasping of meaning. To see, hear, or smell implies seeing *something*, hearing *something*, smelling *something*. Through our bodily interaction with the world, it is always already meaningful for us. Even when we seem to perceive something that is meaningless, such as listening to two people talk in a language that we don’t understand, this situation still has meaning for us. We understand it, namely, as a conversation between two people. Perhaps we can also understand something of the nature of the conversation, if they e.g., seem to be having a heated argument, or if one of them is trying to romantically seduce the other. What we see, listen to, or understand is never completely without meaning, never meaning*less*. Or, put differently, even the meaningless has meaning, *as* meaningless.

In addition, the French word *sens* has a meaning that the English word fails to capture, namely *direction*. One can for example speak of *dans le bon sens* when talking about *the right direction*. This meaning of the world *sens* is important for Merleau-Ponty, since it shows how meaning is not something which we have acquired, but rather something which we *aim at*. Meaning is never fulfilled, but keeps evolving. Gadamer’s (1995/1960) understanding of understanding shows precisely this. Already when we start to read a text, we have an initial understanding of the whole text, of what it is going to tell us. We are thus *directed to* the meaning of the text, already jumping ahead or anticipating what it might tell us (something which Gadamer terms “preunderstanding”). This meaning evolves while we read the text, and is never finished, not even when we have read the last page. The next day we might read another text that changes our understanding of what we read the day before. Meaning is thus *both* something that we have already understood, and something that keeps evolving. Our existence can be understood as taking place between this *already understood* and *understanding anew*.

For Merleau-Ponty, all of these meanings of *sens/sense* are connected. Meaning defines our way of relating to the world, through our senses, in an anticipating way, always prefiguring a whole phenomenon that keeps developing and changing as our perception and understanding changes and develops. Meaning is thus not something that belongs to the object perceived, but neither is it something which the subject bestows upon the object. It arises from the interaction between subject and object, from a human engagement in a world that belongs to us and that we belong to. The world is meaningful thanks to our projects in it, so that the glass of water is not a thing amongst other things, but rather something which we can take hold of and drink from while thirsty, or throw with force in the floor when angry. As human beings, we are as much grasped by meaning as we grasp meaning. Understanding meaning is thus not a cognitive act—we do not deduce it out of given clues, but it rather takes hold of us—like when we smell freshly brewed coffee or recognizes a friend in a crowd of people. Only in afterthought can I examine that which I have already understood, and how I have understood it.

## Understanding meaning in research

The challenges that come with understanding meaning in research do not arise because this is a very difficult or complicated operation, but rather the opposite. Merleau-Ponty’s philosophy shows us how we, as human beings, are always already involved with understanding meaning. It is in fact such an everyday matter, it is so “natural” and immediate for us, that we just carry on with it even though our research demands of us another kind of openness. It is in fact harder to *not* understand (too quickly or too carelessly) than it is to understand (Dahlberg, 2017; Dahlberg et al., 2008).

The philosophy of meaning that we have briefly presented has several consequences for research. These consequences will now be elaborated upon by the help of four questions: 1. The question of openness to meaning 2. The question of content, 3. The question of description versus interpretation, and 4. The question of method.

### The question of openness to meaning

As noted above, being human and being involved in human activities imply that we have both already understood and are on our way to new understanding. This is of vital importance for research. Being in this process of understanding where we always already have understood something, places a huge responsibility on the researcher in terms of openness, so that we do not take for granted what it is that we see and understand. Researchers may not simply rush forward assuming that understanding is just an everyday matter of fact.

We have previously elaborated on the act of *bridling* as the ability to slow down and reflect on the process of understanding, so that we don’t understand too fast or too carelessly (Dahlberg et al., 2008). Bridling is thus essentially a sort of self-reflection, a continuous investigation of one’s own point of departure, one’s presumptions and presuppositions. By working with our habits of perceiving and knowing, we can stop them from working blindly, that is, from jumping ahead to this or that taken-for-granted understanding. By means of reflecting on, and asking questions to our own understanding (e.g., What is it that I understand? Why is it that I understand in this way?), it is possible to understand differently.

It is important to note that even though the act of bridling implies a heightened self-awareness, it is made in order to become more attentive to the phenomenon one is investigating. The goal of bridling is to reach that presence where we are open for the new; an improvisational openness where we don’t know what will show up but are attentive and ready for it.^1^ Instead of fixating upon a taken-for-granted understanding, bridling helps us to open our understanding to more possibilities. To be a researcher thus means to consciously dwell in the place of *not knowing* rather than knowing.

### The question of content

The analysis above shows how the question of meaning is essential to any qualitative research approach. With phenomenological philosophy in general and Merleau-Ponty in particular, we have shown how there is no “pure” content to operate within qualitative research: meaning is always there. Studies that describe their method as content analysis prove this themselves by using concepts such as ”meaning units”, ”latent content” and ”interpretation”. We don’t argue that such a method cannot be used at all, we simply ask of authors who wish to apply content analysis to be clear about how they understand, and thus use, concepts such as meaning and interpretation, and what philosophical or other theoretical framework they refer to. Since meaning tends to slip in through the back door when one is intent upon content, researchers (or students) who use content analysis should explain the difference between content and meaning, and they must furthermore do that on their own since this is not articulated by content analysis descriptions.

### The question of description versus interpretation

In the aforementioned study (Dahlberg & Dahlberg, 2019) we conclude that there are no philosophical arguments for the ambition to separate “description” from “interpretation”, as is often done in contemporary phenomenological and hermeneutical empirical research. We show, for example, how the idea of inseparability between man and world, subject and object, is a common ground both for phenomenology and hermeneutics. Husserl, as well as Heidegger, Gadamer and Merleau-Ponty, explain why and how we must be aware of that we are *of* the world and that the world is *of* us, and how this foundational inseparability is the birthplace of meaning. This means that understanding is both a process that has always already started (we have always already understood something), and at the same time it is a process that is never finished (we are continuously understanding anew). Meaning keeps evolving.

If we transfer this understanding of meaning as defining our relationship to the world to empirical research, “description” cannot be a matter of stating what people say in e.g., interviews. Description in qualitative research is, inevitably, description of meaning. Understanding meaning in empirical research thus includes both the laying out of intentional meaning, often called descriptive analysis, *and* a kind of analysis that moves beyond present data including external material, often called interpretive analysis. Instead of fighting about how to label one or another kind of analysis, the most important issue in any kind of analysis is how to embody the open and reflective awareness of how meanings come to be and are best articulated.

Theory can be a useful tool in qualitative research, in particular in relation to multi-faceted or ambiguous existential phenomena, which otherwise are not fairly understood or even go unnoticed. Theory can also be important in order to recognize and articulate social structures or power relations that otherwise are hard to identify. There is, however, an epistemological challenge in introducing theory in empirical research. Put short, all well-formulated theory or research results have strong voices that easily silence the less articulated voice of the lifeworld. We therefore advise researchers (or students) not to include external data, such as theory or previous research results, from the start. Before including any kind of external data, which easily dictates the analysis, it is necessary to work with empirical data with an open mind, until an, at least provisional, meaning structure has emerged. There should thus be a description of the investigated phenomenon before theory is introduced. The meaning structure should give a fair clue of what the phenomenon is like, what characterizes the phenomenon and makes it this very phenomenon and not another one (often called “essential meanings”), and it should also inform the reader of the other characteristics of it, i.e., meanings that are more or less dependent on a particular context (often called “particular” or “individual meanings”).

Describing a phenomenon as the result of an empirical analysis involves presenting a structure of meanings, or in other words, meanings that are related to each other. Disparate categories, which are sorted in a column or line, don’t serve a meaning oriented approach well.

If the study is a part of a student assessment, a meaning structure, based on empirical data only, could very well be the final outcome of the work. The alternative that a theory is included in some way is also thinkable. However, for the lower levels of university education, we propose that students include the theoretical information in the discussion part of the paper. If the assessment is at master or doctoral level, the analysis could proceed further, including theory or previously published research results of the phenomenon.

### The question of method

The philosophy of meaning that we have presented has consequences also for the understanding of what method is in empirical research. The understanding of method as a standardized procedure reduces research to the passive adherence to a scientific instruction manual. Instead, we argue in favour of an approach to the question of method where the researcher’s open and bridled attitude is the most important feature. This “method idea” is therefore actually more of a non-method than a method in the ordinary sense.

This open approach to research is guided by the quest of meaning, that is, by revealing the phenomenon that is the object of research. Already in the preparation of research questions and aim, the focus should be on meaning, i.e., on a phenomenon, and the quest of understanding a phenomenon directs how to design a study, including methods of data gathering/generation as well as data analysis.

For us, a “method” consists of different research tools that one can use depending on the aim of the research. There are thus tools that are better than other. If e.g., focus is on lived experiences, interviews are often better than questionnaires. Observations work better with non-verbal phenomena as well as with situations or cultures, and narratives may be a good choice when a life-span needs to be portrayed. The characteristics of the phenomenon and its meanings also direct which tools to use in the analysis. All these choices must be seriously thought through. And they must be guided by an open, meaning oriented approach that is at work all through the research. If the researcher is not able to embrace such attitude, there is no tool that can replace it.

What then, do we answer students that ask for standardized methods, or that find philosophy too difficult or obscure? Can we ask of research beginners to orient themselves in the research landscapes with nothing but the open approach that we advocate to guide them?

To understand philosophy can be a challenge, and even more so when it comes to applying philosophical ideas in empirical science. Not everyone, however, has to travel the same roads, but can learn from those who have been there. We propose that students or other beginners read texts by scholars who have worked with philosophy and shown its relevance for empirical research (e.g., Dahlberg et al., 2008; Vagle, 2014; Ashworth, 2015; Karlsson, 1987, Todres 2007). There are furthermore philosophers who present epistemological and ontological ideas in a more accessible manner, so that it may function as guide for researchers (e.g., Zahavi, 2019). Of course, different scholars focus on different philosophical themes and concepts. However, we argue that regardless of what scholar one chooses to learn from, this work should provide a reflection on how meaning is unavoidable, how meaning originates, and thus how one as researcher relates to meaning.

Students or researchers who do not want to engage with the original philosophy can also learn from published empirical research experiences, how methods have been chosen and skilfully used by researchers in their field. The presentation of epistemological and methodological arguments and considerations in these works can inspire others in their projects. One example is a rewarded Dutch thesis that has proved to challenge the practise of a proposed law with the aim to assist and support people with a wish to die (van Wijngaarden, 2016). The law indicates that the choice of signing up for “assisted suicide” is a rational act. However, by explicating the lived experience of those who have made this choice, the research shows a phenomenon that is far more complex. This researcher presents thorough epistemological and methodological arguments and considerations, which can support other researchers or students. Here one can learn about the question of meaning and how an open attitude can be practiced, as well as see the importance of such an epistemological attitude.

There are many other studies that illustrate epistemological and methodological arguments and choices, e.g., why and how the research design benefits from philosophical insights and how empirical results can be further explored by theoretical analysis grounded in philosophy (e.g., Hörberg & Dahlberg, 2015, Karlsson & Sjöberg, 2009; Lindberg, Ekebergh, Persson, & Hörberg, 2015, Palmér, Carlsson, Brunt, & Nyström, 2014). Further, there are studies where the researchers have expanded and synthesized their previously published empirical results into new theoretical ideas and e.g., clinical implications (cf. Berglund, 2014; Ekebergh et al. 2018).

## Conclusions

The question of meaning is indeed a momentous issue, in several ways. That we, as Merleau-Ponty (1995) puts it, are *condemned to meaning*, implies that meaning is our fate; it is the way in which we relate to our world and thus we cannot flee from meaning. But the question of meaning is critical also in another way. The fate of qualitative research depends on how well we can respond to this question, since it is the rationale for there being qualitative research at all. Furthermore, the employment of content analysis and other standardized methods, where the origination of meaning is not accounted for, cannot respond to the quest of revealing complex, multi-faceted knowledge of existential phenomena in health care or other human science areas. They are thus of poor quality, show less validity and the outcomes therefore show less evidence (cf. Dahlberg, 2017; Dahlberg & Dahlberg, 2019; van Wijngaarden, van der Meide, & Dahlberg, 2017).

The reason why the question of meaning has become fatal, is, as we see it, that researchers try to escape meaning in the name of pragmatism. Understanding meaning is thought to be too complicated or too philosophic. The reason why the question of meaning is challenging in research, is however something quite different. As human beings, we relate to meaning constantly. The challenge is thus not *how* to understand meaning, but rather how to not understand too quickly.

We have also argued that not every researcher has to study Husserl or Foucault. Researchers and students can also use and learn from scholars and researchers who have worked with philosophy in order to make it applicable for empirical science. Furthermore, we also propose a change in the view of how science is best learned. It is now time to strengthen the position of theory of science in university education. If we want our students to understand the *what, why* and *how* of science and research, we have to offer foundational insights into the history of science; how our understanding of science and its criterions for excellence have originated and how it has developed. We cannot any longer be blind to where the positivist movement has brought us. We must establish the fact that scientific research never simply can be about “how to apply a method”, neither in research education nor in lower levels of education. All research benefits from profound insights into ontological, epistemological and methodological questions. For us in qualitative research, this is obvious, but also quantitative researchers have to relate to theory of science, in order to understand the question of evidence as well as the question of meaning. No number, no scale or other quantity—and no word—can stand on its own. All seriously dedicated researchers must understand the need to know what statistics as well as words *mean*.

## Note

1. Bridling as improvisational openness is elaborated on in a forthcoming publication (Dahlberg in press).
